# Single-Scaffold Genome Sequence of Probiotic Strain Bifidobacterium breve BR03 (DSM 16604), Obtained by Combining Hybrid Sequencing and Optical Mapping

**DOI:** 10.1128/MRA.00241-19

**Published:** 2019-05-16

**Authors:** F. Fracchetti, A. Del Casale, M. Tebaldi, A. Cunego, I. Campedelli, E. Salvetti, D. Ederle, F. Deidda, M. Pane

**Affiliations:** a Open Innovation Department, Microbion Srl, Verona, Italy; b BioLab Srl, Novara, Italy; University of Maryland School of Medicine

## Abstract

Bifidobacterium breve BR03 (DSM 16604) is known for its health-promoting activity. We present a single-scaffold genome obtained by using a hybrid approach combining long- and short-read sequencing techniques integrated by an optical map. This approach could be set as an industry standard for probiotic strain characterization.

## Abstract (precis)

Bifidobacterium breve BR03 (DSM 16604) is known for its health-promoting activity. We present a single-scaffold genome obtained by using a hybrid approach combining long- and short-read sequencing techniques integrated by an optical map.

## ANNOUNCEMENT

Bifidobacterium breve BR03 (DSM 16604) was isolated from the feces of a healthy infant. It is a safe strain that is well characterized for its health-promoting activity and already available on the market for human consumption. Probiotic effects of B. breve BR03 include gut colonization (1), the ability to restore the abundance of some microbial communities and to improve evacuation disorders and hard stools (2), inhibitory activity toward Gram-negative bacteria (3), anti-inflammatory activity which stimulates *in vitro* the intestinal cells to produce interleukin 10 (IL-10) (4), and alleviation of muscle tension after muscle-damaging exercise (5).

Here, we report a single-scaffold finished genome sequence of *B*. *breve* BR03 obtained with a combination of two different sequencing technologies (hybrid whole-genome sequencing and optical mapping) to be proposed as an industry standard for probiotic strain identification and differentiation.

*B. breve* BR03 was grown anaerobically overnight at 37°C in de Man-Rogosa-Sharpe (MRS) broth plus cysteine (Oxoid), and the genomic DNA was extracted using the Wizard genomic DNA purification kit (Promega), according to the manufacturer’s recommended protocol for Gram-positive bacteria. DNA quantification was performed using a NanoDrop Lite spectrophotometer (Thermo Fisher Scientific); 25 μg of DNA was used for PacBio and Illumina sequencing.

A standard PacBio library preparation protocol was followed; a size selection was performed to select insert fragments in the range of 3 to 4 kb on average; the library was sequenced on one single-molecule real-time (SMRT) cell. The data collected were processed and filtered using the SMRT Analysis software suite. The continuous long read (CLR) data were filtered by read length (>50), subread length (>50), and read quality (>0.75). The sequencing yielded 117,300 reads (329 Mb) with an average length of 2,811 kb and a GC content of 57.11%. The data collected from the Illumina MiSeq platform using Nano v2 chemistry with 250-bp paired-end (PE) reads yielded 1,148,758 reads. The quality of the Illumina FASTQ sequences was enhanced by trimming off low-quality bases using the “Trim sequences” option in CLC Genomics Workbench v7.0.4. PacBio and Illumina Reads were assembled, scaffolded, and closed using the “De novo assembly” option in CLC Genomics Workbench v7.0.4 (the optimal k-mer size was automatically determined using KmerGenie [6]), SSPACE-LongRead scaffolder v1.0 (7), and GapFiller v1.10 (8). Default parameters were used for all softwares unless otherwise specified. The assembly was validated through a whole-genome map (WGM) by OpGen, which was generated with a KpnI restriction enzyme, giving 420 fragments, of which 194 fragments were smaller than 3 kb (the threshold defined for data filtering [9]). Unweighted pair group method using average linkages (UPGMA) map distance dendrograms were calculated from WGM consensus map data using the MapSolver v3.2.4-PH (OpGen Technologies, Inc.). The combination of these methods yielded one scaffold made of 30 contigs for a total of 2,274,532 bp (*N*_50_ value, 361,038 bp), which were ordered using *B*. *breve* UCC2003 as a reference (GenBank accession number CP000303). The genome was then manually finished through PCR amplification and Sanger sequencing.

### Data availability.

The project has been deposited at DDBJ/EMBL/GenBank under the accession number CP034770. The version described in this paper is the first version. PacBio raw reads were deposited at the Sequence Read Archive under the accession number PRJNA512062.

## References

[1] Mogna L, Del Piano M, Mogna G 2014 Capability of the two microorganisms *Bifidobacterium breve* B632 and *Bifidobacterium breve* BR03 to colonize the intestinal microbiota of children. J Clin Gastroenterol 48:S37–S39. doi:10.1097/MCG.0000000000000234. 25291125

[2] Del Piano M, Carmagnola S, Anderloni A, Andorno S, Ballarè M, Balzarini M, Montino F, Orsello M, Pagliarulo M, Sartori M, Tari R, Sforza F, Capurso L 2010 The use of probiotics in healthy volunteers with evacuation disorders and hard stools: a double-blind, randomized, placebo-controlled study. J Clin Gastroenterol 44:S30–S34. doi:10.1097/MCG.0b013e3181ee31c3. 20697291

[3] Mogna L, Del Piano M, Deidda F, Nicola S, Soattini L, Debiaggi R, Sforza F, Strozzi G, Mogna G 2012 Assessment of the *in vitro* inhibitory activity of specific probiotic bacteria against different *Escherichia coli* strains. J Clin Gastroenterol 46:S29–S32. doi:10.1097/MCG.0b013e31826852b7. 22955353

[4] Drago L, De Vecchi E, Gabrieli A, De Grandi R, Toscano M 2015 Immunomodulatory effects of *Lactobacillus salivarius* LS01 and *Bifidobacterium breve* BR03, alone and in combination, on peripheral blood mononuclear cells of allergic asthmatics. Allergy Asthma Immunol Res 7:409–413. doi:10.4168/aair.2015.7.4.409. 25749784 PMC4446640

[5] Jäger R, Purpura M, Stone JD, Turner SM, Anzalone AJ, Eimerbrink MJ, Pane M, Amoruso A, Rowlands DS, Oliver JM 2016 Probiotic Streptococcus thermophilus FP4 and Bifidobacterium breve BR03 supplementation attenuates performance and range-of-motion decrements following muscle damaging exercise. Nutrients 8:642. doi:10.3390/nu8100642. PMC5084029 27754427

[6] Chikhi R, Medvedev P 2014 Informed and automated *k*-mer size selection for genome assembly. Bioinformatics 30:31–37. doi:10.1093/bioinformatics/btt310. 23732276

[7] Boetzer M, Pirovano W 2014 SSPACE-LongRead: scaffolding bacterial draft genomes using long read sequence information. BMC Bioinformatics 15:211. doi:10.1186/1471-2105-15-211. 24950923 PMC4076250

[8] Boetzer M, Pirovano W 2012 Toward almost closed genomes with GapFiller. Genome Biol 13:R56. doi:10.1186/gb-2012-13-6-r56. 22731987 PMC3446322

[9] Bosch T, Verkade E, van Luit M, Pot B, Vauterin P, Burggrave R, Savelkoul P, Kluytmans J, Schouls L 2013 High resolution typing by whole genome mapping enables discrimination of LA-MRSA (CC398) strains and identification of transmission events. PLoS One 8:e66493. doi:10.1371/journal.pone.0066493. 23805225 PMC3689830

